# Properties of alternative microbial hosts used in synthetic biology: towards the design of a modular chassis

**DOI:** 10.1042/EBC20160015

**Published:** 2016-11-30

**Authors:** Juhyun Kim, Manuel Salvador, Elizabeth Saunders, Jaime González, Claudio Avignone-Rossa, Jose Ignacio Jiménez

**Affiliations:** Faculty of Health and Medical Sciences, University of Surrey, Guildford GU2 7XH, U.K.

**Keywords:** metabolic engineering, microbiology, modularity, *Pseudomonas*, whole-cell models

## Abstract

The chassis is the cellular host used as a recipient of engineered biological systems in synthetic biology. They are required to propagate the genetic information and to express the genes encoded in it. Despite being an essential element for the appropriate function of genetic circuits, the chassis is rarely considered in their design phase. Consequently, the circuits are transferred to model organisms commonly used in the laboratory, such as *Escherichia coli*, that may be suboptimal for a required function. In this review, we discuss some of the properties desirable in a versatile chassis and summarize some examples of alternative hosts for synthetic biology amenable for engineering. These properties include a suitable life style, a robust cell wall, good knowledge of its regulatory network as well as of the interplay of the host components with the exogenous circuits, and the possibility of developing whole-cell models and tuneable metabolic fluxes that could allow a better distribution of cellular resources (metabolites, ATP, nucleotides, amino acids, transcriptional and translational machinery). We highlight *Pseudomonas putida*, widely used in many different biotechnological applications as a prominent organism for synthetic biology due to its metabolic diversity, robustness and ease of manipulation.

## Introduction

The goal of synthetic biology (SB) is to design and build organisms endowed with a set of pre-defined technical specifications. Great computational and molecular efforts take place to assemble the components of genetic circuits and metabolic pathways, typically encoded in DNA vectors, which are delivered to a suitable cellular host for expression known as the chassis. There are a number of potential hosts for the carefully crafted genetic material, and these organisms are selected according to the task that needs to be accomplished considering a variety of factors. These factors include the ability of the chassis to survive the environmental conditions of the final application, its metabolic properties, and the availability of a versatile array of molecular tools for manipulation. Although genetic circuits are designed following engineering principles such as those of modularity and orthogonality — lack of potential interactions between the heterologous material with other components of the chassis — the chassis itself is normally considered a mere vessel and it is not taken into consideration in the design phase [1].

In this review, we explore some additional properties that could allow the chassis to become an integral part of SB designs. Deep engineering of a chassis is of utmost importance in other disciplines, such as those related to transport of goods and people, in which the chassis is devised to serve not one, but multiple applications. For instance, in the aerospace industry, the versatility of the chassis is a key feature, and the same model of aeroplane must allow for accomplishing several and sometimes unrelated tasks: transport of passengers and goods in different configurations, surveillance, fire-extinguishing, support of communications, etc. A good example of a chassis being designed to accommodate a subset of modules is the project Ara for building smartphones. The project aims to generate an open platform for the production of phones with longer lifespans and adaptable to new hardware developments, which is also likely to reduce waste generation since the modules are replaceable (http:://www.projectara.com).

**Figure 1:**
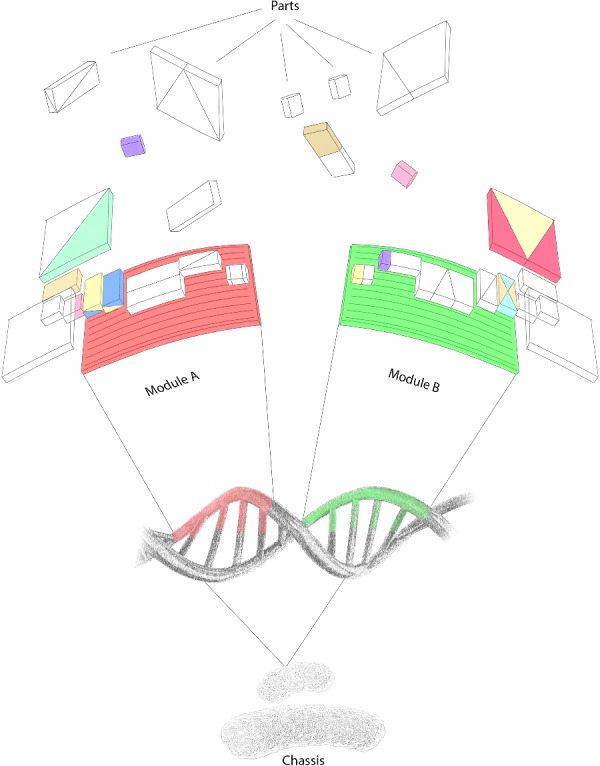
Engineering modular chassis The notion of modularity has been widely and successfully used in the context of genetic circuits. On the basis of the current state of technological development on whole genome synthesis and editing, we envisage the design and construction of modular chassis that could be easily modified to serve not a single but multiple purposes.

A working definition of an ideal microbial chassis could be that of an organism capable of supporting the activity of the engineered exogenous genetic components for as long as required without interfering with their original purpose. Although it is not restricted to them, the analogy of the chassis in SB is often applied to the micro-organisms commonly used in biotechnology. Some properties sought in microbial chassis are robust growth, robust cellular envelope, simple transcriptional and translational control, well-defined metabolic networks and absence of evolutionary processes that could affect the performance of the exogenous circuits [2]. Similar to the examples from other technological disciplines, it is possible to think of approaches targeting the development of a chassis from first principles that could be used in a variety of applications (Figure 1). In practical terms, this would translate into a microbial generalist that could be easily adapted to different functions. Generalists can dominate populations subject to environmental fluctuations [3]; however, as soon as an ecosystem becomes more stable, evolutionary trade-offs take place to maximize the fitness of the organisms. As a result, stable ecosystems are largely dominated by specialists adapted to defined conditions [4]. This has as a consequence that most of the organisms currently used as potential chassis in SB have already become specialists and are selected for further modification in the laboratory depending on how close they are to the final application.

Alternatively, different groups of researchers are working on a bottom-up strategy trying to characterize microbes containing the minimal set of genetic information to support life as vehicles for biotechnological applications. Pioneering works in this direction were carried out in *Escherichia coli* [5] and current efforts also include organisms belonging to the genus *Mycoplasma* [6,7]. These *Mycoplasma* species have undergone severe reductions in their genome, will then be used as ‘blank canvasses’ to insert additional functions [8]. However, these species have specialized during evolution to become symbionts, hence they have lost many of the functions required to sustain rapid growth and produce large amounts of biomass as desirable for a SB chassis [7].

## Chassis used in synthetic biology

The most commonly used organisms in SB are the bacteria *Escherichia coli* and *Bacillus subtilis* and the yeast *Saccharomyces cerevisiae.* The main reason for their popularity is that these are model organisms widely studied in the laboratory and for which an ample catalogue of molecular tools is available. It is possible to characterize and predict their physiology very accurately, benefitting also the understanding of the performance of SB circuits and pathways. Despite possibly not being the most suitable hosts, they have been successfully used in many applications [9–11].

Table 1 contains a comprehensive list of other bacterial species that are of biotechnological interest and used to different extents in SB. They cover a range of both Gram-positive and Gram-negative bacteria that have been traditionally used for fermentations, production of secondary metabolites, enzymes and vaccines, as well as probiotics. They all meet the definition of a chassis, although they have been selected because they are endowed with some unique properties that make them ideal for a potential function, rather than for universal/general applications. Hence none of these single chassis would be considered the best for SB, but rather, the best so far for carrying out each function in particular. They are all considered model organisms in their respective fields and have in common that they can be engineered at some level, in addition to a set of molecular tools and whole-genome models being available. Despite the diversity, there are a number of lessons that can be learned from them.

**Table 1.** Alternative chassis used in Synthetic Biology applications

Organism	Applications	Lifestyle	Reference
*Clostridium acetobutylicum*	Biofuels, flavouring, cosmetics, plasticisers	Anaerobic	[67]
*Klebsiella* spp.	Biofuel production	Facultatively anaerobic	[68]
*Pseudomonas putida*	Bioremediation, small molecules and bioplastics production	Aerobic	[69]
*Streptomyces* spp.	Antibiotic, secondary metabolite and protein production.	Aerobic	[70,71]
*Shewanella oneidensis*	Electricity production	Facultatively anaerobic	[72]
*Geobacter sulfurreducens*	Electricity production	Anaerobic	[72]
*Bacteroides thetaiotaomicron*	Control of gut microbiota	Anaerobic	[73]
*Synechocystis* spp.	Biofuels, small molecules production fixing CO_2_	Photosynthetic	[74]
*Deinococcus radiodurans*	Bioremediation and small molecule production under stress	Aerobic	[75]
*Mycoplasma* spp.	Minimal genome engineering and vaccine	Facultative anaerobic	[7]

## Desirable technical specifications of a chassis inspired by biology

### Lifestyle

The lifestyle of a particular organism is one of the main reasons for selecting it as a chassis. Chemoheterotrophs, such as *E. coli*, are typically used for production of small molecules [9] and materials [12] because of their fast and inexpensive growth. They can use a wide variety of substrates for growth from glucose to waste products giving them the added value of their use as agents for removal of pollutants in a closed economy. In addition, they can use a number of final electron acceptors and the species used in SB also range from the strict aerobic (like some pseudomonads) to the strict anaerobes that ferment or use inorganic molecules or anodes as final acceptors, making them interesting organisms for bioelectrochemical processes such as production of bioelectricity [13]. Photoautotrophs are also relevant organisms for SB due to their innate capability of fixing atmospheric CO_2_ and produce other C-based compounds. Cyanobacteria such as *Synechocystis* and the commonly known *Spirulina* — a mixture of *Arthrospira platensis* and *Arthrospira maxima* — have been engineered for production of biofuels and other commodity chemicals [14]. They are hypothesized to be key to a future manned mission aimed at colonizing Mars that would also involve the use of methanogenic and plastic-producing bacteria [15]. A different lifestyle useful for SB is that of some *Clostridium* [16] and *Geobacter* [17] species. They are capable of taking energy from the electric current of an anode and fixing CO_2_, potentially allowing the linking of renewable sources of electricity (wind, solar, etc.) to the production of fuel in a process known as microbial electrosynthesis [17,18].

### Cell envelope

The cell envelope is another important factor. Micro-organisms are often exposed to harsh environments where they must cope with high shear forces and other physical stresses [2], therefore they are endowed with a robust cellular envelope. For certain SB applications, this cell envelope must also allow modifications, for instance, to secrete proteins to the extracellular medium or to decorate the surface of the cell with antibodies used to engineer attachment and tropism [19]. It may be required that this cell envelope is devoid of many components (proteins, polysaccharides, etc.) in, for example, applications requiring the display of proteins and their recognition by the immune system, such as production of live vaccines using *Mycoplasma pneumoniae* (http:://www.mycosynvac.eu).

### Chassis–circuit interactions

An important factor contributing to the performance of exogenous genetic circuits is their interplay with the host [20]. A number of cellular functions are affected due to the presence of the newly inserted genes. These genes can fail to be expressed at high levels because of the presence of over-imposed regulatory events such as catabolic repression. It is therefore crucial to have a good understanding of the network of interactions within the chassis before implementing new functions. Even in the absence of specific regulatory events, there is competition between the endogenous genes and the synthetic pathways taking place for the transcriptional and translational resources [21], among other cellular capabilities. Current efforts aim at understanding the factors constituting this so-called metabolic burden [22] so that genetic circuits can be designed more efficiently, for instance by insulating some of their shared components [23]. In this way, their impact in the host can be predicted. Although these efforts have produced a picture of the availability of resources in *E. coli*, further work is required to determine whether the same distribution laws apply to all organisms and what will be the interplay between circuits and cellular machinery in other chassis.

### Metabolic network

Similarly, in the case of genes encoding metabolic pathways, the correct implementation of an external pathway should consider the interactions with the endogenous metabolic network. This can be achieved by generating whole metabolic reconstructions based on genome information data [24]. These reconstructions are later manually and experimentally refined, and the performance of the modelled organism tested through flux balance analysis (FBA). In a typical FBA simulation, gene expression is not considered and this limits the applicability of the method to cases where steady-state assumptions hold. Despite this, FBA can still be very informative and can highlight modifications required in the metabolic network to optimize a process, for example by knocking-out genes encoding competing pathways [24]. However, the highly non-linear nature of metabolic networks makes this a non-trivial exercise, and other pathway analysis tools are required for successful genetic manipulation.

**Figure 2:**
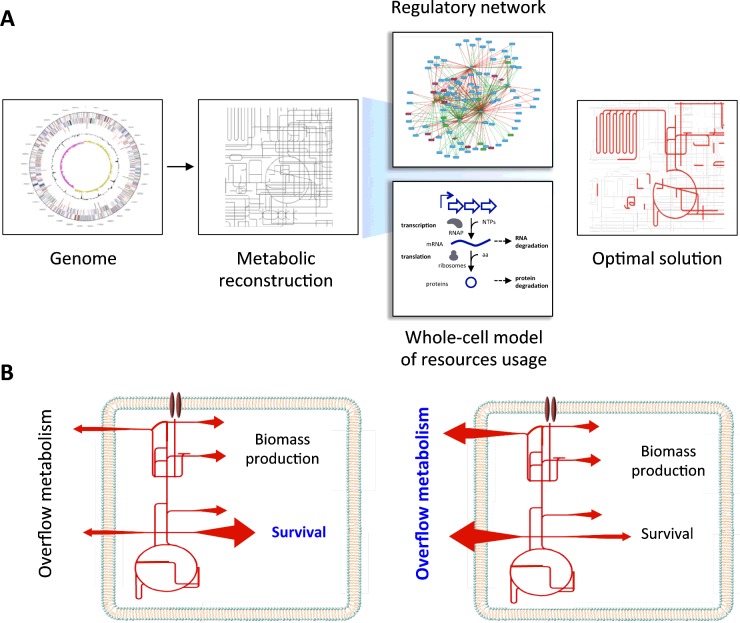
Engineering the interplay between exogenous circuits and the metabolism of the chassis (**A**) Scheme depicting current methods of obtaining whole-cell models from genomic information. Metabolic reconstructions are constrained by incorporating networks of regulatory interactions and the investment of cellular resources (e.g. ATP, nucleotides, amino acids, RNAP and ribosomes) in the transcription and translation of the enzymes responsible for the metabolic reactions. (**B**) Extremophilic bacteria are endowed with a versatile metabolism that allows investing energy and resources in the mechanisms needed to survive harsh conditions (left-hand panel). In the absence of environmental stressors those resources are invested in overflow fluxes that could be capitalized for the production of alternative products (right-hand panel).

In order to produce more accurate predictions beyond the static view of the network, dedicated methods of analysis are then required to constrain FBA models to known parameters of the chassis, for example, the levels of gene expression or the availability of cellular resources for transcription and translation [25,26]. The combination of these computational methods can then lead a dynamic representation of the status of the cell that could be applied to any chassis (Figure 2A). The interplay of the heterologous circuits and the host could then be captured by the value of the intracellular concentration of certain molecules, for example ribosomes or, as other authors suggest, ATP and NAD(P)H [27]. In an ideal chassis these values should be maximized, although bacteria are very efficient at adapting the level of resources to their needs as a way of preventing misuse and waste of energy. In an attempt to optimize resources expenditure, many laboratories around the world have taken part in genome minimization projects. The underlying hypothesis is that, by removing unneeded genes, the cost of maintenance of the cell will be lower and therefore there will be more resources available for the synthetic circuits and pathways. This approach has been successfully validated in *E. coli* [5] among others, making genome plasticity another desired feature of an ideal SB chassis.

Related to this, accurate dynamic predictions will help to unveil other metabolic properties that could be of interest in a chassis. The so-called ‘overflow metabolism’ [28,29] is one of these properties in which SB can have a significant effect. The ‘overflow metabolism’ generates a waste of carbon and energy by synthesizing by-products when certain pathways are saturated (e.g. the formation of acetate by *E. coli* when glucose consumption exceeds a threshold). Overflows are avoided in metabolic engineering and they can be overcome by incorporating the appropriate activities as illustrated by the decrease in acetate production in *E. coli* when expressing a recombinant NADH oxidase to tune the redox ratio [30]. Metabolic overflows can be useful if they are redirected into the synthesis of a compound of interest as demonstrated by the production of alanine from glucose in *E. coli* [31]. In this example, a double mutant in the H^+^-ATPase and lactate dehydrogenase can direct an excess of pyruvate synthesis into alanine production through the expression of a recombinant alanine dehydrogenase from *Geobacillus stearothermophilus*.

The metabolic network of micro-organisms adapted to extreme environments offers properties amenable to have included in an ideal chassis. It is well established that protein production is the most expensive cellular process in *E. coli* accounting for the consumption of 74% of ATP. In addition to the protein production burden, extremophiles have to spend vast amounts of energy to cope with the harsh environments that they colonize. For example, organisms living in environments with high concentrations of salts need to synthesize ‘compatible solutes’ [32,33], representing a high energetic and metabolic cost [34]. The concentrations of these intracellular solutes can be rapidly adjusted in response to the external conditions, so that micro-organisms using this strategy can often adapt to live in fluctuating environments [35]. As a consequence, these organisms are capable of producing a ‘surplus’ of metabolites (i.e. ATP) that, in the absence of the environmental stress, lead to a metabolic overflow [36]. This is illustrated in the production of ectoine(s) in the halophilic bacteria *Chromohalobacter salexigens* (Figure 2B). When this species is cultured under low osmotic pressure, therefore not requiring compatible solutes for survival, it produces a burst of ATP that can be used in other processes if properly channelled [36].

It is also worth mentioning, as reviewed recently in [37], that the perfect chassis may not be a single organism, but rather a microbial community due to the benefits of social interactions. Some of these benefits include better resistance to harsh environments and toxic compounds, as well as the possibility of labour distribution, which may alleviate the burden on each cell by sharing substrates and products of synthetic pathways with other members of the community.

## The case of *Pseudomonas putida*

The bacterium *Pseudomonas putida* KT2440 has been proposed as a suitable chassis for SB projects [38]. This soil bacterium is renowned in the field of bioremediation because of its metabolic diversity, especially regarding its ability to degrade aromatic compounds [39]. It was certified by the FDA (U.S. Food and Drug Administration) as a ‘safe’ micro-organism and its popularity is also reflected in the number of molecular tools that have been initially designed for its manipulation, which in most occasions can be applied in other bacterial species benefiting the whole community of applied microbiologists [40]. To complete the set of tools, and thanks to both the availability of a well-annotated genome sequence [41,42] and extensive experimental validation, the genome-scale reconstructed metabolic network model of *P. putida* KT2440 has been constructed and analysed through FBA [43,44].

**Figure 3:**
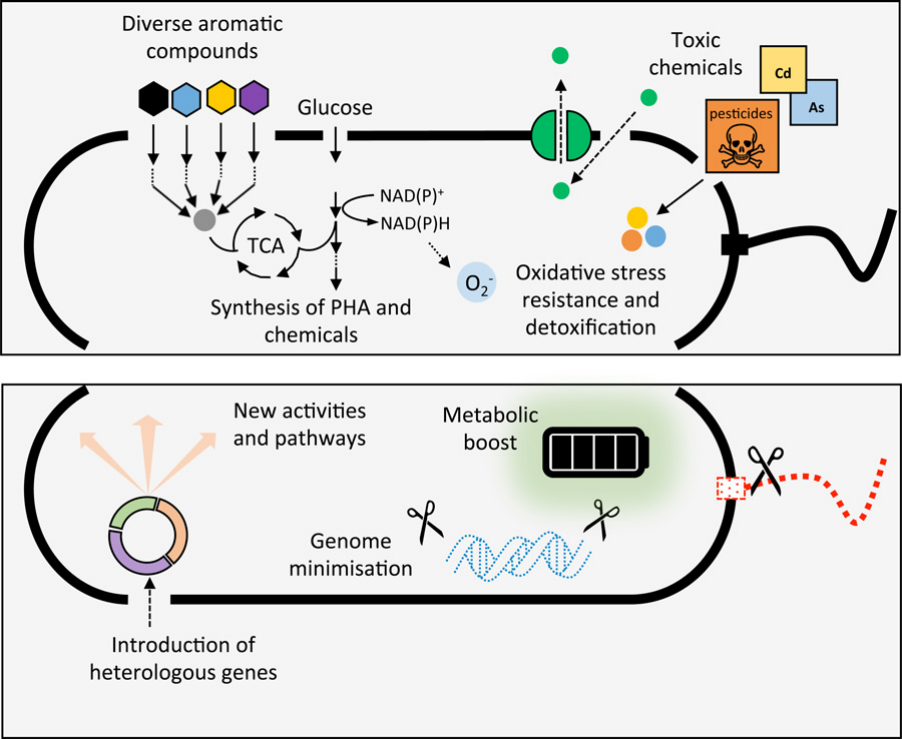
*Pseudomonas putida* as a suitable chassis for multiple applications in synthetic biology *P. putida* is naturally endowed with outstanding capabilities for biodegradation, biotransformation, bioplastic production, detoxification and stress survival (upper panel). The strain KT2440 has been upgraded further using synthetic biology with a dedicated set of molecular tools and genome editions to boost its metabolic capabilities further (lower panel).

In addition, *P. putida* also combines several of the properties discussed above, making it a versatile chassis for synthetic biology (Figure 3). *Pseudomonas* species are adaptable to challenging selective pressures and can quickly develop resistance to various drugs [45]. *P. putida* also contains multiple efflux pumps contributing to their ability to resist toxic compounds [46]. The combination of those two properties makes *P. putida* a good candidate for laboratory directed evolution experiments aimed at the production of toxic metabolites through the expression of synthetic pathways. Furthermore, *P. putida* can cope with high oxidative stress thanks to the assimilation of glucose through the Entner–Doudoroff pathway along with activities of the incomplete Embden–Meyerhof–Parnas and the phosphate pentose pathways (EDEMP cycle) [47]. This metabolic feature constitutes an example of overflow metabolism: the EDEMP cycle produces a higher concentration of reducing equivalents and this has been hypothesized as being the reason behind the diverse catabolism for aromatic compounds displayed by *P. putida* KT2440 [48,49].

These metabolic properties have been exploited in a series of applications. The strain KT2440 is still a major agent used in the mineralization of toxic aromatic compounds such as toluene and naphthalene through plasmid-encoded enzymes [50,51]. By adding the relevant genes, this battery of activities can be expanded to deal with the environmental accumulation of several xenobiotics and pesticides such as methyl parathion and hexachlorocyclohexane [52], as well as for the detoxification of cadmium [53] and arsenic [54].

In addition to biodegradation applications, *P. putida* is also used as a cell factory in SB. A major breakthrough is the expression of antibodies which production seems to be restricted to only heavily engineered *E*. *coli* strains. In that sense, the synthesis of single-chain Fv fragments [55] as well as camel antibodies (nanobodies) [56] has been achieved in *P. putida* with good yields and proper folding. Apart from proteins, *P. putida* has been used to produce a wide number of small molecules such as rhamnolipids, terpenoids, polyketides and non-ribosomal peptides (recently reviewed in [57]).

*P. putida* KT2440 can also naturally accumulate polyhydroxyalkanoates (PHAs) to high yields [58], even under industrial-scale production conditions [59], making this strain an ideal candidate for commercial synthesis of biopolymers with thermoplastic properties. This natural mechanism can be tuned to produce plastics with very specific properties, for instance changing the length of the side chain length of the polymers [60]. Another very important factor is the diversity of the carbon source that *P. putida* can use for PHA production. Some strains of *P. putida* can synthesize PHAs using polystyrene [61] or vegetable oil waste [62] as substrates. This opens the gates to the potential application of *P. putida* for plastic recycling, contributing to solve the problem of the environmental fate of plastics (http:://www.p4sb.eu) [63].

Besides the many applications of *P. putida* KT2440 discussed above, there is room for its improvement as a superior chassis for SB. By carrying out directed genome minimization, it has been possible to obtain versions of the strain with a reduced metabolic burden that can achieve greater heterologous gene expression [64]. Finally, although *P. putida* KT2440 is not a good producer of biofilms, it is possible to manipulate the intracellular concentration of messengers such as cyclic-diGMP to increase biofilm production resulting in the generation of artificial consortia with enhanced biodegradative properties [65]. This shows that a poor biofilm producer can actually be a good choice for community engineering according a defined set of technical specifications.

## Conclusion

The expansion of SB from traditional laboratory organisms such as *E. coli* to others, depending on the biotechnological application sought, has allowed the identification of key features that make a good chassis. These properties include a suitable lifestyle, fast growth and a versatile envelope, well-characterized regulatory events, the possibility of engineering social interactions, and an accurate map of its metabolic network. By modifying all of these properties, the streamlining of organisms such as *P. putida*, which is now considered a reliable chassis for SB applications, has been possible. In addition to desired technical specifications, we have identified the need for obtaining reliable predictions of the physiological status of the cell by combining metabolic models with models accounting for availability of resources and their investment. The dynamic descriptions of the cell obtained this way could then be used to select the chassis more efficiently and also to highlight important metabolic modules needed for a certain application.

Despite these features having been identified in many organisms, *E. coli* continues to be the preferred bacterial chassis for SB projects. In fact, recent developments such as the modification of the *E. coli* lifestyle to express the Calvin cycle [66] are close to our vision of the design of a modular chassis. In this vision, the chassis would be designed from first principles as a collection of genetic modules encoding the functions (structural, metabolic, etc.) most appropriate to sustain the desired activity. These modules would be implemented in a minimal organism capable of self-propagation and fine-tuned through laboratory-assisted evolution. Whether this minimal chassis should be inspired by *E. coli* exclusively, a different organism or a collection of them, is part of the current discussion in the field and will be determined by technological advances and future applications of SB.

## Summary

- Organisms used as workhorses in synthetic biology, typically referred to using the term “chassis”, are not taken in consideration in the design phase of applications.
- Despite being suboptimal, model host organisms, such as *Escherichia coli*, are often selected for these applications, mainly due to a good understanding of the biology and the availability of molecular tools.
- The concept of modularity can also be applied to the design of an efficient and versatile chassis by endowing a suitable host with certain properties.
- Properties that need to be considered are lifestyle, endurance to environmental stress and well-characterized regulatory and metabolic networks. The knowledge about the physiology of the organism must allow for the use of predictive global models accounting for the usage of resources (metabolites, ATP, nucleotides, amino acids, transcriptional and translational machinery) and potential interactions with exogenous circuits.
- *Pseudomonas putida* is an organism that possesses many of these properties. It is a robust and versatile biocatalyst that can be easily manipulated, constituting an ideal candidate for numerous applications in synthetic biology.

## Abbreviations

EDEMP cycle: Entner–Doudoroff, Embden–Meyerhof–Parnas and pentose phosphate pathways
FBA: flux balance analysis
PHA: polyhydroxyalkanoate
SB: synthetic biology
